# Mesenchymal stem cells for treating ocular surface diseases

**DOI:** 10.1186/s12886-015-0138-4

**Published:** 2015-12-17

**Authors:** Liyun Zhang, Vivien Jane Coulson-Thomas, Tarsis Gesteira Ferreira, Winston W. Y. Kao

**Affiliations:** Department of Ophthalmology, University of Cincinnati, Ohio, USA

## Abstract

Mesenchymal stem cells (MSC) have become a promising tool for cell therapy in regenerative medicine. They are readily available, demonstrate powerful differentiation capabilities and present immunosuppressive properties that aid them in surviving from host immune rejection for its great potential use in allograft. Currently clinical trials are underway using MSC, both culture-expanded allogeneic and autologous, for the treatment of a range of diseases not treatable by conventional therapies. A vast array of studies has dedicated towards the use of MSC for treating corneal diseases with very promising outcomes. MSC have successfully differentiated into keratocytes both *in vitro* and *in vivo*, and corneal epithelial cells *in vitro*, but it is uncertain if MSC can assume corneal epithelial cells *in vivo*. However, to date few studies have unequivocally established the efficacy of MSC for treating corneal endothelial defects. Currently, the diversity in protocols of the isolation and expansion of MSC are hindering to the assessment of cell treatment ability and the further development of treatment regimens. Therefore, future studies should develop international standards for MSC isolation and characterization. In this review, we discuss recent advances in MSC for treating ocular surface diseases.

## Introduction

Mesenchymal stem cells (MSC) are a group of fibroblast-like multipotent mesenchymal stromal cells [1, 2]. They were originally identified as multipotent stromal precursor cells in bone marrow by Friedenstein and his co-workers in 1970s [3–5]. The name of MSC was first introduced by Caplan in 1991 [6] who found these cells attained multipotent characteristics and could differentiate into multiple distinctive specialized cells. Since the stemness of MSC was potentially useful for treating diseases [2, 7, 8], MSC attracted the attention of many researchers. Besides from the bone marrow, MSC are also found in many other connective tissues, such as umbilical cord [9], adipose tissue [10] and corneal stroma [11, 12]. MSC have been isolated, cultured and characterized in various ways by numerous investigators, which makes it hard to compare the cell properties and the treatment outcomes obtained from different laboratories. In light of these discrepancies the Mesenchymal and Tissue Stem Cell Committee of The International Society for Cellular Therapy has suggested a minimal criteria to define the MSC: MSC are plastic-adherent, must present a certain surface molecule profile (markers) and be able to differentiate to a characteristic tri-cell lineage, i.e., osteoblasts, adipocytes and chondrocytes *in vitro* [1, 13].

Most of MSC studies draw attention to their therapeutic efficacy, which have been extensively conducted in many body systems and organs, such as central nervous system, heart, blood, lung, liver, kidney, pancreas, joint, skin and eye, etc. [2]. The application of MSC in ocular diseases was superbly summarized in elegant reviews by Joe et al. [14] and Yao & Bai [15] and Li and Zhao [16]. The former mainly focused on the efficacy of treating retina degeneration, uveitis and glaucoma optic neurophathy, while the latter two focused on corneal reconstruction. In this review, we will summarize the characterization of MSC and discuss the advance of MSC research made in treating cornea and other ocular surface diseases, e.g., dry eye diseases.

### Identification and characterization of MSC

Like many other cell types, MSC isolated from tissues are able to adhere to the plastic surface of cell culture dish and propagate *in vitro*. They are fibroblast-like and express certain cell surface markers, though no single marker or a set of markers can be simply applied to define MSC. Multiple characterization tests must be performed and the combined results are used to identify the MSC thereby avoiding misclassification.

### MSC immunophenotype

Immunophenotype analysis is one of the essential tests for MSC. In general, the minimum cell surface molecules that should be examined include positive markers: CD105 (endoglin), CD73 (5'-nucleotidase) and CD90 (Thy-1); and negative markers: CD45, CD34, CD14, CD11b (integrin αM chain), CD79α, CD19 and HLA-DR surface molecules [13]. Many other markers have also been suggested to be indicative for the identification of MSC, such as the expression of CD13, CD29, CD44, CD106, CD166, and the lack of CD38, CD31 [17]. Stem cell-related transcription factors, such as Nanog, Oct-4 and Sox-2 [18], are also helpful in characterizing MSC. Fluorescence-activated cell sorting is routinely conducted to evaluate the purity of cell population.

### MSC differentiation capacity

The MSC multipotent capacity was usually assessed by their multilineage differentiation into several mesenchymal tissues. The capacity of tri-cell-lineage differentiation, i.e., osteogenesis, adiposegenesis and chondrogenesis is the gold standard for identifying MSC and any cell preparation must meet this minimum requirement prior to being classified as MSC [13]. For such, MSC are cultured in a specific induction medium for 2 to 3 weeks in order to induce differentiation into the specific cell types. Thereafter, stainings for calcium, lipids and proteoglygans are performed to show whether the cells have been functionally specialized into osteocytes, adipocytes and chondrocytes, respectively. The potential of MSC for neural differentiation [17] and cardiogenesis [9] have also been used as criteria in some studies, however, this is not used as a routine method.

### Others

There are other tests that may be employed for estimating the function of MSC. Colony-forming unit-fibroblast (CFU-F) assay [19] is useful to quantify the colony generation capacity of MSC. Cell growth kinetics measurement can refelct the cell expansion ability. Cytokine expression spectrum is also a means of evaluating the secretion ability of MSC. MSC have been found to secrete SCF, LIF, M-CSF, Flt-3, IL-6, GM-CSF, G-CSF, SDF-1/CXCL12 and VEGF, however, not IL-3 [17].

### Characteristics of MSC from different tissues

MSC were initially isolated from the bone marrow [5, 19, 20]. Thereafter, many other tissues were found to contain MSC, such as umbilical cord [9, 21, 22], placenta [23], adipose tissue [10, 24], skeletal muscle [25, 26] and dental pulp [27]. The relatively abundant tissue sources and easy isolation procedures make MSC an excellent option of stem cells for autologous and allogeneic application in treating diseases. Studies have shown that different tissue origins provide advantages and disadvantages in terms of their future clinical application. The bone marrow was the first MSC source to be investigated and is still the most abundantly studied. However, the isolation of bone marrow MSC (BMMSC) requiring the invasive aspiration from donor greatly restricts its application. Wharton’s jelly isolated from human umbilical cord is a rich source of MSC that can be easily expanded and stored in liquid nitrogen for immediate use [9]. MSC derived from the umbilical cords (UMSC) are believed to be more primitive than cells obtained from adult tissues. Moreover, umbilical cords are plentiful and usually discarded as biological waste. Recently, the adipose tissue is becoming a popular source for MSC isolation. The adipose tissue is another rich source of MSC (ATMSC) and enables auto-graft.

Whether MSC isolated from different sources present similar properties is an important question since it may determine which is most suitable for treating specific disease(s). Several comparison studies have been performed toward this objective [28–32]. Morphology and cell marker analysis to date has not identified significant differences among MSC isolated from bone marrow, umbilical cord blood (UCBMSC) and adipose tissue [28]. However, their colony generation, proliferation and differentiation capacities are not equal. For example, the colony generation frequency is different with the highest in ATMSC and lowest in UCBMSC. The proliferation capacity is the highest in UCBMSC and the lowest in BMMSC. In comparison to the BMMSC, UCBMSC have higher osteogenic ability but lower adipogenic potential [29]. ATMSC have higher chondrogenic potential than UMSC from Wharton jelly [30], but lower osteogenic potential than BMMSC [31]. Additionally, different MSCs present distinct immune modulatory capabilities [32]. ATMSC have been shown to be the most effective in inhibiting the differentiation of monocyte-derived dendritic cells when compared to BMMSC [32]. However, MSC from BM, Umbilical Wharton’s jelly and AT present no differences in inhibiting phytohemagglutinin-induced T-cell proliferation. So far, it is not clear if these discrepancies provide any insight into which MSC treatment would be the most appropriate for treating various diseases.

### Transdifferentiation of MSC to various corneal cell types

MSC can give rise to a variety of mesodermal cells as described above, and also have transdifferentiation ability to assume phenotypes of neural ectodermal cells and epithelial cells [33]. Furthermore, it has been shown that BMMSC could resemble limbal fibroblast cells which assist in maintaining the limbal epithelial stem cells in the limbal niche [34]. Both BMMSC and limbal fibroblasts show a highly similar gene expression profile, including CD106, CD54, CD166, CD90, CD29, CD71 and CD105. In addition, BMMSC and keratocytes all express CD13, CD29, CD44, CD56, CD73, CD90, CD105 and CD133 but not HLA-DR, CD34, CD117 and CD45 [35]. These studies suggested a possibility that MSC can be guided to differentiate towards corneal cells. Nevertheless, *in vivo* there is a lack of direct evidence to substantiate the differentiation of MSC to assume corneal epithelial cell phenotypes. Although, the differentiated cells *in vitro* could be used in corneal tissue engineering or cell replacement treatment. In Table 1, we summarize the current studies on MSC transdifferentiation towards corneal cells types (Table 1).

**Table 1:** Summary of the studies on MSC differentiating into corneal cells

Cornea cell Differentiation	MSC type	*In vitro*		*In vivo*		Reference
		Method	Differentiation test	Method	Differentiation test	
Epithelium	Human BMMSC	No	No	Rat alkali burn model received BMMSC on AM	Human Krt3 (−); human keratin-pan (−)	[37]
	Rabbit BMMSC	Coculture with Rab-LSC or Rab-LSC conditioned medium	Krt3 (+)	Rabbit alkali burn model received BMMSCs on fibrin gel	Krt3 (+)	[38]
	Rat BMMSC	Coculture with rat corneal stromal cell	Krt12 (+)	Rat alkali burn model received induced MSCs on AM	Clinical assessment; Krt12 (+)	[39]
	Human ATMSC	Coculture with basal culture medium conditioned with human corneal epithelial cells	Krt3 (+); Krt12 (+)	No	No	[40]
	Human BMMSC	Sphere culture treated with RA, BMP4 and EGF followed by the cell dissociation and Matrigel culture	Krt3 (+); Krt12 (+); Krt8(+); Transepithelial Electrical Resistance test	No	No	[41]
Keratocyte	Human UMSC	No	No	*Kera−/−* mouse and *lum−/−* mouse received UMSC corneal injection	Human keratocan (+); Lumican (+); CD34 (+); ALDH3A1 (+)	[44]
	Mouse BMMSC	No	No	*Kera−/−* mouse received BMMSC corneal injection	Human keratocan (+)	[45]
	Human BMMSC	Cultured in human keratocyte conditioned medium	Human keratocan (+); Lumican (+); ALDH1A1	No	No	[46]
Endothelium	To be studied	To be studied	To be studied	To be studied	To be studied	

This table lists all the references of studies on the MSC differentiating to all corneal cell types

*BMMSC* bone marrow derived mesenchymal stem cell, *ATMSC* adipose tissue derived mesenchymal stem cell, *UMSC* umbilical cord derived mesenchymal stem cell, *Krt3* keratin 3, *Krt12* keratin 12, *Krt8* keratin 8, *AM* amniotic membrane, *Rab-LSC* rabbit limbal stem cell, *ALDH1A1* aldehyde dehydrogenase 1 family member A1

### Corneal epithelial cells

During development, the corneal epithelium derives from the surface ectoderm [36]. Whether MSC can be reprogrammed to cells of ectodermal lineage has been investigated. Early experiments reported that the MSC transplanted onto cornea do not transdifferentiate into epithelial cells *in vivo* [37]. In this study, human BMMSC were seeded on amniotic membrane and sutured on the chemically injured rat cornea. BMMSC could survive and repress the cornea inflammation, but failed to undergo corneal epithelium differentiation determined by CK3 expression [37]. However, a later study carried out in rabbits inclined to draw a positive conclusion [38]. BrdU labelled BMMSC were placed on fibrin gels and transplanted onto the alkali burned cornea. These BrdU positive cells participated in the cornea healing and were found to express CK3, implicating BMMSC differentiated into corneal epithelial cells.

The outcome of many *in vitro* experiments supported the idea that MSC are able to assume cornea epithelial cell phenotype under certain conditions, however to date *in vivo* data has shown contradictory results. The first *in vitro* experiment described was performed by co-culturing rabbit BMMSC with corneal limbal stem cells (LSCs) or LSC conditioned medium [38]. The BMMSC were found to change morphology from fibroblast-like to the broad and flattened epithelial shape in both culture systems. The immunofluorescence staining and flow cytometry analysis identified transiently increased CK3 expression in BMMSC. Jiang et al. subsequently reported that corneal stromal cells also have the similar ability to induce BMMSC to become epithelial cells. They seeded these cells on amniotic membrane and transplanted them onto the cornea of limbal stem cell deficient rats. The results showed that corneal neovascularization was significantly reduced by the transplantation of epithelium equivalent seeded on amniotic membrane. It is surprising to note that UMSC-derived epithelium equivalent yielded a better outcome than that of the direct transplantation of MSC seeded on amniotic membrane. Why the differentiated epithelium is more effective in neovascularization repression and ocular surface reconstruction deserves further investigation [39]. After co-culture with corneal stromal cells, ATMSC exhibited epithelial cell morphology and expressed the corneal epithelial cell marker CK12. Furthermore, the authors examined if the differentiated cells presented corneal epithelial cell biological function. Recently, adipose tissue derived ATMSC were shown to attain the ability to differentiate into the corneal epithelium. After culture in corneal epithelial cell conditioned medium for 15 days, ATMSC switched their morphology to epithelial-like and up-regulated Krt12 expression [40]. Even though diverse groups have described the differentiation of MSC into corneal epithelial cells, the precise mechanism remains elusive.

A recent investigation has revealed a few factors which may contribute to the MSC transdifferentiation. In the study by Katikireddy et al. [41], BMMSC were induced to assume ectodermal cell types by culturing in 3-dimensional spheres in medium containing retinoic acid (RA), bone morphogenetic protein-4 (BMP-4) and epidermal growth factor (EGF). The expression of p63 and CK8 of mRNAs were measured to indicate successful transdifferentiation. Moreover, it was found that MSC that are positive for stage-specific embryonic antigen-4 (SSEA4), an early embryonic stem cell marker [42], have higher potential to differentiate into corneal epithelial cells than SSEA4 negative MSC. The SSEA4+ MSC expressed higher levels of stem cell markers, such as Sox2, Oct4, Nanog, Rex1, ABCG2 and TRA-1-60, and can be further induced to present epithelial cell morphology and express corneal epithelium specific molecules, i.e., CK3 and CK12. The epithelium barrier integrity test, trans-epithelial electrical resistance (TER), showed the cells induced from SSEA4+ MSC present a 2-fold increase in barrier integrity than SSEA4- cells. However, they did not get to the normal TER range of corneal epithelial cells. Certainly, further optimization of the induction conditions and a longer follow up may help to confirm the possibility of obtaining functional epithelium from MSC.

### Corneal keratocyte

Keratocytes are derived from the periocular mesenchyme cells of neural crest origin [43]. Successful differentiation of umbilical and bone marrow MSC into keratocytes was performed in animal studies [44, 45]. In these experiments, DiI-labeled BMMSC and UMSC were transplanted into mouse cornea stroma under disease conditions. One to two weeks after the surgery, MSC became dendritic and expressed keratocyte specific proteins, KS-keratocan (keratan sulfate keratocan) and KS-lumican.

*In vitro* differentiation study further confirmed the *in vivo* result [46]. When BMMSC were cultured on amniotic membrane nourished with keratocyte-conditioned medium, they quickly exhibited dendritic cell shape, within 24 h. Moreover, they produced keratocan, lumican and aldehyde dehydrogenase 1 family member A1 (ALDH1A1). It was thought that some secreted factors from the keratocytes were essential for MSC differentiation to corneal stromal cells. However, no critical factor that promotes such cell fate change was identified in this study.

### Corneal endothelial cells

Only two previous studies were related to the potential of umbilical mesenchymal stem cells and bone marrow mesenchymal stem cells in differentiation to assume the corneal endothelial cell phenotypes, Joyce and coworkers showed that hUMSC could adhere to the denuded corneal endothelium and assume corneal endothelial cell like phenotypes in an *ex vivo* culture model [47]. Liu and Zhao showed that in a rabbit model autologous BMMSC transplanted on denuded corneal endothelium became irregular in shape similar to corneal endothelial cell [48]. However, the characteristics and functions of transplanted UMSC and BMMSC were not rigorously examioned.

### Therapeutic application of MSC

The application of MSC for treating various dysfunctions in different systems has been extensively studied and reviewed [2, 49, 50], including applications in the eye [14]. Currently, there are about 200 ongoing clinical trials registered in the National Institute of Health public database http://clinicaltrials.gov. The treatments cover a wide variety of diseases, such as bone/cartilage diseases, immune/autoimmune disorders, heart diseases, gastrointestinal diseases, neurodegeneration and diabetes. Relatively few MSC clinical trials have focused on ocular diseases. However, many animal studies have been dedicated towards exploring the therapeutic potential of MSC for treating retinopathy, uveitis, glaucoma and ocular surface disorders [14]. Cornea is an immune privileged tissue and its external location and transparency allows easy assessment facilitating the evaluation of the therapeutic efficacy in live animal after MSC transplantation making it a valuable model for MSC studies. Our lab and other labs have reported that MSC transplantation could treat both congenitally diseased corneas and chemically damaged corneas (Table 2).

**Table 2:** Summary of the studies on MSC in treating corneal diseases

Cornea anomalies		Species	Application	Reference
Inherited cornea anomalies	Lumican null	Mouse	Intrastromal injection	[44]
	MPS IIV	Mouse	Intrastromal injection	[57]
Cornea Chemical burn		Mouse	Intrastromal injection	[58]
		Rat	Cornea surface transplantation	[37]
		Rat	Topical application	[59]
		Rat	Subconjunctival injection	[60]
		Rabbit, rat, mouse	Systematically application	[63–65]
Persistent cornea epithelium defect		Human	Cornea leision injection	[66]
GVH dry eye		Human	Blood infusion	[67]

This table summarizes the current research on MSC treating corneal diseases both in animal and human. The MSC application methods are specified

*PMS IIV* Mucopolysaccharidosis type VII

### Animal studies

- Congenital corneal diseases

### Lumican null mice

Lumican (Lum) is a member of keratan sulfate proteoglycans which belongs to the small leucine-rich proteoglycan family [51]. It is expressed as a glycoprotein in most connective tissues, while the cornea presents the proteoglycan form, containing KS side chains. KS-Lum is primarily synthesized by keratocytes in corneal stroma and plays a major role in maintaining the corneal transparency by regulating the collagen fibrils assembly. The *Lum*^*−/−*^ mice present thin and opaque corneas due to the irregularly spaced and thickened collagen fibrils which results from the lack of keratan sulfate proteoglycans (KSPG) [44, 52, 53]. The phenotype displayed by these mice serves as a model for general congenital disorders which involve corneal opacities due to irregularities in collagen arrangement.

Our group found that human UMSC were able to effectively treat the corneal opacity of these mice [44]. In this study, both human UMSC and umbilical cord-derived hematopoietic stem cells (UHSC) were intrastromally transplanted into the *Lum*^*−/−*^ mouse corneas. Only UMSC but not UHSC transplantation improved the corneal transparency. The cornea thickness increased and the collagen fibers were re-organization enabling the corneas to become transparent. The UMSC presented reduced proliferation after transplantation and morphologically resembled the dendritic keratocyte. Moreover, they expressed keratocyte specific proteins, such as keratan sulfate proteoglycans, KS-keratocan and KS-lumican. Follow-up observations showed that injected UMSC which were labeled with DiI were present in the cornea for at least 3 months. The relatively long-term survival of xenografted UMSC was supposedly made possible by the immune modulatory ability of the MSC. This was supported by the immunostaining results in which less infiltration of leukocytes and macrophages were seen in UMSC transplanted corneas when compared to those transplanted with UHSC.

### Mucopolysaccharidosis type VII (MPS VII) mice

MPS VII, also known as Sly syndrome, is a lysosomal storage disease. It is an autosomal recessive inherited disease caused by a mutation in the *GUSB* gene coding β-glucuronidase [54–56]. The deficiency of the β-glucuronidase enzyme impedes the catabolism of heparan sulfate, dermatan sulfate and chondroitin sulfate at the glucuronic acid residues leading to the accumulation of glycosaminoglycans (GAGs) in lysosomes, which affects a multiple tissues and organs, such as the brain, bone and eye. The MPS VII mouse corneas exhibit a cloudy appearance. Our group showed that UMSC transplantation significantly reduced the cornea opacity [57]. The total GAG content in treated corneas decreased approximately 30 %, when compared to the untreated corneas, reaching levels similar to the littermate control mice. The lysosomal-associated membrane protein 2 (LAMP2) staining manifested that the number and size of lysosomes in keratocytes drastically decreased throughout the treated corneas when compared to the untreated littermate controls. This study unveiled that UMSC were able to secrete exosomes which spread throughout the entire cornea and were up-taken by both host keratocytes and endothelial cells. These observations strongly indicated that the intercellular trafficking between UMSC and host cells contributed to the catabolism of GAG enabling lysosomal recycling in the diseased cornea. It is very likely that these vesicles carry endoglycosidases which enable the turnover of the accumulated GAGs in host keratocytes, endothelial cells, and extracellular matrix.

b. Chemical burn

Corneal chemical and thermal burns are common eye traumas. The injured area and the severity can vary a lot with damage ranging from only limited on the ocular surface causing epithelium wounds, corneal stroma opacity and/or neovascularization, to much more severe which penetrates into the eye leading to persistent intraocular inflammation and destructions. In the corneal alkali burn rat model, human BMMSC were seeded onto an amniotic membrane which was then sutured on the cornea surface [37]. This treatment regimen successfully aided corneal epithelium regeneration, and at the same time suppressed the corneal neovascularization. The rats vision was improved as determined by behavioral assay. The immunostaining for cell markers and cytokines indicated that the treatment efficacy of MSC was due to their ability to suppress inflammation. MSC survived on the cornea surface for at least four weeks after the transplantation. Nonetheless, they did not assume corneal epithelial cell phenotype.

We have recently shown that UMSC transplanted into the alkali burnt mouse cornea suppress the immune response enabling recovery of a transparent cornea within 2 weeks, while control mice present severe inflammatory response as this same time-frame [56]. Moreover, we further unveiled that the UMSC secrete a specific glycocalyx which traps and suppresses the immune cells [58].

In another study of ethanol burned rat corneas, it was found that both MSC and MSC conditioned media applied 3-times per day were able to reduce the cornea inflammation and neovascularization, in turn increased the corneal transparency [59]. The inflammatory response assay showed a reduction of CD4^+^ T cells infiltration into the treated cornea accompanied by reduced secretion of pro-inflammatory cytokines, e.g., IL-2 and IFN-γ, while anti-inflammatory cytokines, e.g., IL-10 and TNF-β increased. This study suggested that the anti-inflammatory and anti-angiogenic effect of MSC most likely relies on their paracrine capacity. This idea was further supported by another experiment of a rat alkali burn model in that the subconjunctival injected MSC promoted cornea wound healing via attenuated inflammation and neovascularization [60]. The results suggested that it is not necessary to transplant MSC in the wounded area to achieve therapeutic effects.

In a study involving chemical burn in rabbit corneas, Rb-MSCs were suspended in fibrin gels and transplanted onto injured rabbit corneas, restoring the corneal surface [15, 38]. These MSC showed expression of cytokeratin 3 (CK3), a corneal epithelial-specific marker. Another *in vivo* study confirmed that MSC have the ability to differentiate into corneal epithelial cells in experimental limbal stem cell deficiency rabbit model, maintaining stem cell characteristics, while some even transdifferentiated into epithelial progenitor cells [61]. Human MSC (hMSC) are also able to survive and migrate into the cornea stroma after transplantation onto the surface of the alkali-burned rabbit cornea, not only differentiating into corneal epithelium cells but differentiating into cells other than epithelia [62].

Furthermore, several groups have shown that the therapeutic effect of MSC in cornea could also be obtained via systemic administration. After the corneal injury, the intravenous or intraperitoneal infusion of MSC all increased the corneal transparency and suppressed the inflammation. These observations were obtained in several experimental animal models, such as rabbit, rat and mouse. However, the opinions on whether the introduced MSC could engraft into the cornea are inconsistent. For example, some studies showed the systemically transplanted cells could home to cornea, since the labeled MSC injected through vein was eventually seen in the injured cornea [63–65]. In contrast, other studies presented evidence that injected MSC could not be detected in cornea, even when using sensitive techniques such as quantitative PCR [64]. Instead, they proposed that MSC treatment effect might be derived from the TSG-6 secreted by MSC, and TSG-6 systematically or locally administration can reciprocate the MSC therapeutic effect.

### Clinical trials: treatment of dry eyes with MSC

The application of MSC in treating cornea diseases has made great strides in animal studies. However, few human clinical trials have been conducted due to the safety concerns. So far, only two clinical studies of MSC in treating ocular surface diseases have been performed with very promising results.

One was a case report in which the ATMSC were found to facilitate the epithelial healing in one persistent sterile corneal epithelial defect patient [66]. This patient had keratoconus and got corneal cross-linking treatment one year before an injury occurred in his eye. After the accident, the cornea epithelial cells failed to regenerate, and accompanied by the underlying stromal opacification and mild conjunctival inflammation. He received many medications including antibiotic, anti-herpetic, anti-fungal treatments, artificial tears and soft contact lens within 7 weeks after the injury. None of these treatments showed any signs of improvement. Then, he consented to try MSC transplantation. Autologous ATMSC were topically injected into the bottom of cornea ulcer. Eleven days after the injection, the area of corneal epithelial defect started to regress. And one month later, the cornea was completely healed. This case is the first report of autologous MSC application in human cornea. The result is consistent with animal studies that revealed MSC do have the ability of facilitating corneal epithelial cell regeneration.

A clinical trial of MSC was designed to treat dry eye disorder associated with chronic graft-versus-host disease (GVHD) [67], which is a common complication of the allogeneic bone marrow transplantation [68]. The GVHD can damage multiple organs and tissues, such as skin, eye, liver, lung and immune system. About half of the patients have dry eye problems after receiving hematopoietic stem cell transplantation [69–73]. MSC have been successfully used to treat severe cases of GVHD in humans [74, 75]. Their treatment efficacy for dry eye was observed by Weng et al. [67, 76]. They recruited 22 GVHD-related dry eye patients and gave them intravenous injection of MSC. Twelve out of 22 patients presented improved clinical symptoms as judged by the dry eye scores, the ocular surface index and the Schirmer test results. The peripheral blood test found the number of CD8^+^CD28^−^ T cells, a subgroup of regulatory T (T_reg_) cells, was higher in patients who responded to MSC treatment than those patients showed little improvement. Thus, it was proposed that MSC may enhance the generation of CD8^+^CD28^−^ T_reg_ cells that further modulate the balance of Th1 and Th2 cell populations in the immune response involved in GVHD [77].

In a another study, a one-week topical application of MSCs lead to an increase in aqueous tear volume and improvement in ocular surface evaluation tests in a dry eye model in rats [78]. These authors demonstrated that topical application of MSCs can decrease inflammation by their anti-inflammatory effects, increase aqueous tear volume and improve ocular surface evaluation tests in a BAC induced dry eye model in rats.

### Mechanism of MSC therapy

MSC have shown treatment efficacy for different types of diseases. The molecular mechanisms of their treatment success remains largely unclear. Several possible mechanisms have been proposed.

### Cell replacement

The classical cell replacement therapy uses MSC to functionally reconstitute the hematopoietic microenvironment of bone marrow [79]. When MSC is transplanted into bone marrow of non-obese diabetic/severe combined immunodeficiency mice, they differentiate into multiple cell types essential for keeping hematopoietic cells primitive in terms of function and phenotype.

In congenital cornea diseases, the therapeutic effect of MSC is at least partially attributed to either the supplementation or substitution of corneal cells. In *Lum*^*−/−*^cornea [44], the introduced MSC assumed keratocytes morphology and function. The transplanted UMSC laid down the essential structural components in cornea stroma such as, lumican and keratocan, which helped to re-organized the collagen fibrils leading to normal corneal thickness and transparency. Similarly, MSC transplanted into MPS VII mice cornea also presented keratocytes phenotype [57]. The transplanted MSC provided the functional metabolic enzymes that allowed the degradation of the accumulated GAGs thereby assisting in the lysosome recycling.

### Paracrine competence

MSC secrete several signalling molecules, such as neurotrophic factors, growth factors or cytokines, which can diffuse in the local tissue environment and interact with the surrounding cells. MSC paracrine ability has been studied in many systems, such as hepatic and central nervous system [80–85].

In the cornea, MSC paracrine effects have been well demonstrated by their anti-inflammation and anti-angiogenesis functions in cornea chemical burn models. Under the injured condition, the transplanted MSC could produce a set of signalling effectors that resulted in a decrease of host pro-inflammatory cytokines, e.g., IL-2, MMP2, IFNγ and increase of anti-inflammatory cytokine, e.g., IL-6, IL-10, and growth factor, e.g., TGFβ [37, 59]. These factors then initiate the downstream signalling transduction and finally repress inflammation. Moreover, MSC also change the levels of many angiogenesis-associated factors in cornea, such as TSP-1, MMP-2 and VEGF, which in turn reduce the neovascularisation [59]. Moreover, our recent study demonstrated that a rich specific glycocalyx secreted by UMSC can trap and inhibit inflammatory cells [56].

### Exosome-mediated intercellular trafficking

Exosomes are microvesicles with diameter about 40 to 100 nm, which originate from the fusion of intracellular multivesicular bodies (MVBs) with cell membrane and are released into the extracellular spaces [86]. They are composed of a bi-layered lipid membrane, proteins, mRNA and miRNA [86, 87]. Exosomes are secreted by many types of cells including MSC [88–90]. The MSC-derived exosomes have been reported to have many important biological functions, such as treating cardiovascular disease [89], ameliorating renal oxidative stress [91] and suppressing VEGF expression in breast cancer cells [92].

Our group detected the exosomes released by the transplanted UMSC in the diseased cornea of MPS VII mice, and also found that these exosomes were able to enter into host corneal keratocytes and endothelial cells [57]. The *in vitro* experiments further discovered that UMSC-secreted exosomes assisted in the recycling process of accumulated GAGS in the lysosomes in MPS VII cells. This study proposed a new mechanism of MSC in treating corneal disease.

### Immunomodulatory ability

One of many amazing functions of MSC is that it can regulate the recipient immune response by modulating the maturation and the function of multiple immune cells, such as myeloid dentritic cells [93], natural killer (NK) cells [94], T cells [95–98] and B cells [99, 100] and macrophages. One good example in eye for this immunomodulatory ability is the treatment effect for GVHD-associated dry eye patients [67]. In patients with treatment effect, the level of CD8^+^CD28^−^ T cells was observed higher than those patients without effect. The *in vitro* co-culture experiment showed that MSC could facilitate CD8^+^ cell to undergo CD8^+^CD28^−^ T_reg_ cell fate, a cell type that may regulate the balance of T-helper 1/T helper 2 activity.

### Tissue homing capacity for treating congenital

MSC administrated systemically can home into a wide range of tissues and exist *in situ* for a relatively long period of time. A non-human primate study reported that the infused GFP labelled MSC was detected in 16 distinct tissues with different cell quantities by quantitative PCR at even 21 months after the infusion [101]. Moreover, the amount of MSC in tissues is higher in animals that received lethal whole body irradiation and hematopoietic support than in the non-conditioned animal. It seems that injury facilitates MSC infiltration into tissues.

A similar observation was made for cornea wound healing process [65]. Q-dot or GFP labelled MSC was intravenously injected into mice that received thermal cauterization in one side of the cornea. MSC homed to the injured cornea but not the naïve contralateral uninjured cornea. This led to a faster corneal epithelium recovery than the untransplanted control mice.

### Future research direction

MSC isolated from different tissues by different techniques may possess different levels of abilities which may directly impact their efficacies on treating various diseases. Thus, optimizing the isolation and propagation procedure is critical to make a more reproducible and homogenous cell preparation. Moreover, since the current minimal criteria to define MSC proposed by The International Society for Cellular Therapy is not able to fully indicate the competence of MSC on treating disease, a therapeutic test is required evaluate the cell quality.

So far, the precise mechanism by which MSC treat diseases remains elusive. Further investigations of how MSC modulate the immune response, communicate with host cells and become resident cells will help to delineate the molecular and cellular basis of utilizing MSC to treat diseases.

As for cell replacement therapy in the cornea, one interesting research direction could be inducing MSC to differentiate into corneal endothelial cells *in vitro*, and then performing the corneal endothelial transplant. It is known that cornea endothelial cells are critical in maintaining cornea transparency. Once damaged in adult humans, the endothelial cells are not able to regenerate [102]. Clinically, corneal endothelial keratoplasty has been performed successfully to provide the functional endothelium. However, this surgery is still limited by the availability of suitable donor corneas. If MSC could be guided to transdifferentiate into endothelial cell, it would provide a plentiful source of endothelium graft in lieu of the whole cornea suitable for transplant.

As described above, the transplanted MSC can survive in the cornea and secretes factors to improve the acute conditions of traumatized cornea. However, how long MSC can provide the regulatory effect is not known. Once the MSC engages into the cornea and becomes the resident cells of the tissue, it may lose the stem cell characteristics and fail to provide further protection to the host. In order to maintain sustainable expression of the certain effectors, a genetic modification of MSC could be a meaningful research direction. Several gene engineering studies have demonstrated that MSC secreting erythropoietin (EPO) [103] or brain-derived neurotrophic factor [104, 105] provided persistent neural protective effect in retina degeneration models. Since MSC can live in the tissue for relatively long time, it can be an excellent gene delivery system for treating congenital mutation of metabolic enzymes to do the treatment.

Another issue is the safety of using MSC. It remains uncertain whether MSC have long term adverse effects on the immune system and whether there is a possibility of inducing tumorigenesis. The future studies may need to clarify these concerns.

Compared to systemic administration, local MSC transplantation may have fewer side effects, especially for corneal applications. The avascular nature of the cornea makes it an immunologically privileged tissue [106] and transplanted cells tend to have high survival rates and reduced host versus graft disease. Moreover, the ocular surface location eases the *in vivo* observation making the cornea a very attractive model for studying the application of MSC both in animal models and humans.
